# Processing SPARQL queries with regular expressions in RDF databases

**DOI:** 10.1186/1471-2105-12-S2-S6

**Published:** 2011-03-29

**Authors:** Jinsoo Lee, Minh-Duc Pham, Jihwan Lee, Wook-Shin Han, Hune Cho, Hwanjo Yu, Jeong-Hoon Lee

**Affiliations:** 1 Department of Computer Engineering, Kyungpook National University, Daegu, Korea; 2 Department of Medical Informatics, Kyungpook National University, Daegu, Korea; 3 Department of Computer Science and Engineering, POSTECH, Pohang, Korea; 4 Department of Computer Science, KAIST, Daejeon, Korea

## Abstract

**Background:**

As the Resource Description Framework (RDF) data model is widely used for modeling and sharing a lot of online bioinformatics resources such as Uniprot (dev.isb-sib.ch/projects/uniprot-rdf) or Bio2RDF (bio2rdf.org), SPARQL - a W3C recommendation query for RDF databases - has become an important query language for querying the bioinformatics knowledge bases. Moreover, due to the diversity of users’ requests for extracting information from the RDF data as well as the lack of users’ knowledge about the exact value of each fact in the RDF databases, it is desirable to use the SPARQL query with regular expression patterns for querying the RDF data. To the best of our knowledge, there is currently no work that efficiently supports regular expression processing in SPARQL over RDF databases. Most of the existing techniques for processing regular expressions are designed for querying a text corpus, or only for supporting the matching over the paths in an RDF graph.

**Results:**

In this paper, we propose a novel framework for supporting regular expression processing in SPARQL query. Our contributions can be summarized as follows. 1) We propose an efficient framework for processing SPARQL queries with regular expression patterns in RDF databases. 2) We propose a cost model in order to adapt the proposed framework in the existing query optimizers. 3) We build a prototype for the proposed framework in C++ and conduct extensive experiments demonstrating the efficiency and effectiveness of our technique.

**Conclusions:**

Experiments with a full-blown RDF engine show that our framework outperforms the existing ones by up to two orders of magnitude in processing SPARQL queries with regular expression patterns.

## Background

### Introduction

In recent years, the Resource Description Framework (RDF) has become the most popular sematic web technology for modeling large collections of data over the web. As a W3C standard model for exchanging data among web data repositories, RDF has been used in a large number of applications such as DBpedia [1], a knowledge-management community of structured information extracted from Wikipedia, or freebase [2], an online social database collected from thousands of sources. In the domain of life sciences as well as bioinformatics, RDF is the common data model for a lot of public online bioinformatics resources [3] such as Uniprot (Universal Protein Resource) [4] or Bio2RDF [5].

An RDF can be considered as a collection of facts in the form of triples (*subject*, *predicate*, *object*) that represent the relationship, indicated by the triple pattern *predicate*, between the *subject* and the *object*. An RDF database can also be represented as a directed labeled graph, called RDF graph, in which the subjects and the objects are the nodes of the graph and the predicates are the edges connecting these nodes. In company with the popular availability of the RDF stores, SPARQL [6], the W3C recommendation query language for RDF, has played an important role in searching and extracting the data from various web knowledge-bases. A SPARQL query is a SQL-like RDF query which mainly consists of two clauses - the SELECT clause and the WHERE clause. The SELECT clause specifies triple patterns that need to be returned as the answer for the query, and the WHERE clause consists of series of triple patterns which can also form a query RDF graph pattern. An example of a SPARQL query for finding a chemical compound that has the name “Tryptophan Synthetase” is:

SELECT ?*x* WHERE {?*x* <hasName> “Tryptophan Synthetase”; ?*x* <hasSubstrate> “Chemical”}.

**Figure 1:**
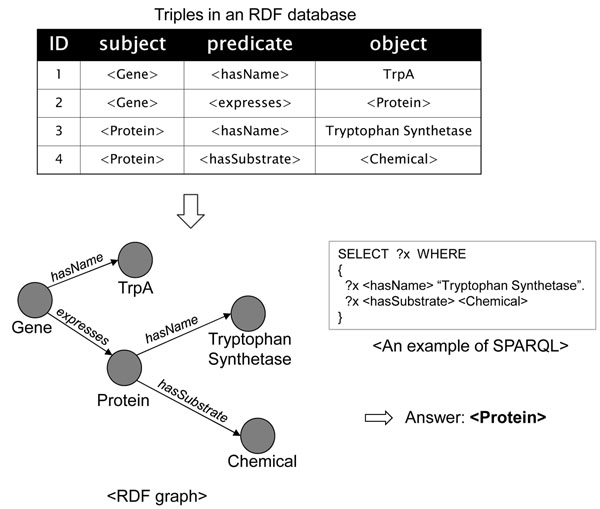
**An example of RDF data and SPARQL query**. Example of the graph representation format of an RDF database, a SPARQL query, and the answer for this SPARQL query.

Figure 1 shows an example of the graph representation format of an RDF database, a SPARQL query, and the answer for this SPARQL query.

In order to express diverse requests on extracting information from RDF data, SPARQL needs to be able to efficiently support regular expression processing. We consider an example where a researcher wants to find all proteins related with the ‘MOUSE.’ While it is hard to express this query without the regular expression, it is desirable and easy to represent this request by using the following SPARQL query with a regular expression.

SELECT ?*protein* WHERE {

?*protein* a <Protein>.

?*protein* <mnemonic> ?*m*.filter(?*m*,“.*MOUSE.*”)

}

Moreover, since users usually do not know the *exact* matching values of an RDF triple pattern, this example presents a common kind of request over RDF data and thus shows the necessity of supporting regular expression processing in SPARQL. It therefore motivates us to study the regular expression processing in RDF systems which, to the best of our knowledge, has not been efficiently supported by any of the existing RDF systems.

The regular expression processing has been studied in many existing literatures [7-12]; however, most of the existing techniques are developed for a given regular expression over a text corpus, not RDF data. Recently, there have been several works dealing with regular expression processing [13-16] in RDF data. However, they merely consider processing the regular expression along the paths between two nodes in an RDF graph, but do not consider processing regular expressions over each RDF triple pattern in a SPARQL query.

In this paper, we propose an efficient framework for efficiently processing the regular expression in the standard SPARQL over an RDF database. Our framework contains two main steps: index building and query processing. In the index building step, we exploit the approach which uses grams (i.e., a sequence of characters of a specific length) in a text corpus [12] for building the index of an RDF data. Specifically, we first extract all the “useful” grams appearing in all the triples of the RDF data. Then, we index all the extracted grams with their occurrence information by using an inverted index structure. In the query processing step, we find all indexed grams in the input regular expression, and then, construct an execution plan using these grams. In order to construct an efficient plan, we propose a cost model for evaluating the plan. To demonstrate the efficiency and effectiveness of our proposed framework, we prototype the framework in C++. Then, by conducting extensive experiments with the real dataset such as GeneOntology [17], we show that our framework can have significant performance improvement over multiple kinds of SPARQL queries supporting regular expression processing.

## Related work

### RDF systems

Along with the growth of Semantic-Web research, there are increasing numbers of studies on RDF - one of the most popular frameworks for representing semantic-web ontologies and knowledge bases. As an RDF database can be considered as a collection of data items in the form of triples (*subject*, *predicate*, *object*), most of the existing RDF systems store the knowledge bases by creating relational tables over these RDF triples [18-26]. They either store all the triples in a giant relational table having three attributes subject, predicate, and object [19], or store each group of triples having the same predicate in a so-called *property* table [22-25]. Early and popular open-source RDF systems such as Sesame [22,23] and [24,25] use the latter method for storing RDF triples. However, these systems have been empirically shown to be unsuitable for large scale datasets [18,19]. The current best performance RDF systems in the approach using property tables, such as Oracle [27] and C-Store-based RDF engine [18], exploit the materialized join views and the indexes on them, and thus, incur the challenge of a physical design problem due to the diversity of predicates and the lack of a global schema. Recently, Neumann et al. [19] has developed a novel RDF system, called RDF-3X, that stores all triples in a huge table indexed by using six compressed clustered B^+^-Trees. By following a RISC-style architecture [28] for indexing as well as query processing, RDF-3X can avoid the problem of physical-design and achieve efficient performance on large join queries - the inherent performance challenge in large RDF dataset. As RDF-3X outperforms other existing systems by a large margin, it can be considered as the-state-of-the-art RDF system. However, as far as we know, there is no RDF system designed for efficiently supporting SPARQL with regular expression pattern.

### Regular expression processing

There is a lot of literature studying the regular expression processing problem [29] which finds the matching strings in a text for a given regular expression (i.e., regex). The most common approach uses the finite automata, in which the given regex is converted to an equivalent DFA, and then, all the strings accepted by the DFA are returned as the results for the regex [7-10]. For efficiently searching the matching strings of the regex in a large amount of documents, many techniques using pre-build indexes for the regex have been proposed. Baeza-Yates and Gonnet [11] construct a suffix trie for indexing all the suffixes of the text and directly simulate the minimal DFA corresponding to the given regex over all paths of the trie in order to find the matching strings. This solution can answer the queries in logarithmic average time; however, since the size of the constructed suffix trie is several times larger than the text corpus, this solution is not suitable for the large databases. In [12], Cho and Rajagopalan speed up regular-expression matching on a large database by proposing an inverted index structure called *multigram* index. In this index, the most “useful” grams such as the most selective grams from the text corpus will be indexed, associating with the posting lists of document IDs containing those grams. Then, a physical access plan containing the indexed grams in the given regex will be generated for facilitating the query processing. However, in contrast to our framework, these regular expression processing techniques are developed for searching over the text corpus, not for querying the RDF data. In the domain of RDF databases, there have been several efforts considering the regular expression processing in RDF queries. However, the existing works concentrate on extending SPARQL queries to process the regular expression over the paths of RDF graphs (i.e., regular paths), while our proposed framework supports the regular expression pattern in the standard SPARQL which is able to process a matching RDF triple pattern for a given regex. Alkhateeb et al. [13,14] adopt a new query language, called PSPARQL, that extends from SPARQL by allowing regular expressions to be used for the labels of the arcs in the query graph. In this query language, the regular expression is used for capturing the relationship information along the paths between two nodes in the RDF graph. Similar to this approach, Kochut et al. [15] propose another extension of SPARQL, called SPARQLer, by using the path variables in the query graph pattern. Here, the path variables are simple paths between two nodes in an RDF graph that contain constraints based on regular expressions. For querying the knowledge graph such as RDF graph in a search engine, NAGA search engine [16] introduces a graph-based query language which is akin to SPARQL that also allows regular expression matching over the paths in the RDF graph, in which the regular expression is placed at the edge label in the query graph.

To the best of our knowledge, there is no work fully supporting SPARQL with regular expression patterns in RDF databases.

## Methods

### Index building

To process regular expressions efficiently, we need to construct an efficient index structure. In this section, we will explain our index structure, and the algorithm for constructing the index.

**Figure 2:**
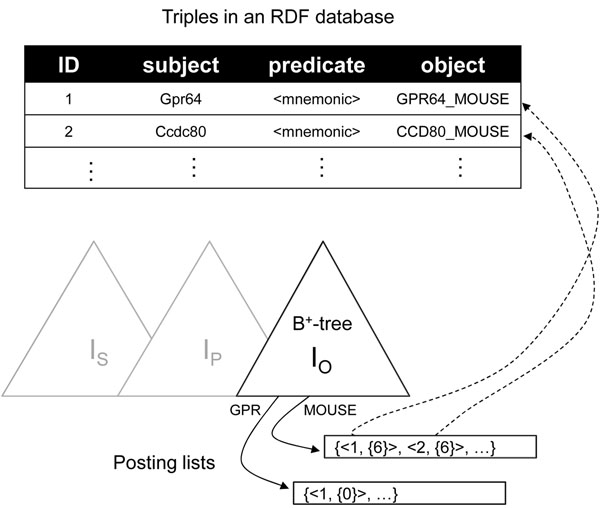
**An example of the inverted index**. There are three inverted indexes for subject, predicate, and object parts of the RDF triples.

First, we formally present some related concepts. Each data item in RDF data is represented as a triple, with the form of (*subject*, *predicate*, *object*). This triple is also called as an *RDF triple*. An *RDF database* is a database storing the RDF data. For a given RDF data, an RDF database ***D*** = {*t*_1_, *t*_2_, …, *t_n_*} is a set of all triples in the RDF data, where *t_i_* is an RDF triple whose ID is *i*. Each RDF triple *t_i_* in *D* contains subject, predicate, and object parts, represented as *t_i_.s*, *t_i_.p*, and *t_i_.o*, respectively. For example, an RDF database for the RDF data in Figure 2 can be represented as

***D*** = {*t*_1_ = (〈*Gpr*64〉, 〈*mnemonic*〉, *“GPR*64_*MOUSE"*), *t*_2_ = (〈*Ccdc*80〉, 〈*mnemonic*〉, *“CCD*80_*MOUSE"*), …}.

Because our index uses the gram as a basic indexing unit, we also need to know the concept of the gram. For a given string *S*, an *n-gram* is a substring of *S* whose length is *n*, and Ξ*_n_* is a set of all *n*-grams of *S*. For example, for the string *S* = *“MOUSE"*, the set of all 3-grams is represented as Ξ_3_ = {*MOU*, *OUS*, *USE*}. In our index building algorithm, for given constants *α* and *β*, we index a subset of  to minimize the index size and maximize the pruning power.

Algorithm 1 shows our index building method IndexBuild. This algorithm returns three constructed indexes ***I_S_***, ***I_P_***, ***I_O_*** using three input parameters, ***D***, *α*, and *β*. The first parameter ***D*** is an RDF database which stores all triples. The other parameters, *α* and *β* are the minimum and maximum size of the grams to be indexed. The algorithm first extracts three strings (*S_S_*, *S_P_*, and *S_O_*), presented in the tree part of the triple (Line 2). Then, for each string, it extracts all grams in the string and stores the selective grams among them by calling the ExtractAndInsertGrams function (Line 3-5). Note that, we build three separate indexes for each subject, predicate, and object parts of triples. The ExtractAndInsertGrams function first finds all *n*-grams, *α* ≤ *n* ≤ *β*, and assigns them to the set ***G*** (Line 7). Then, for each gram *g* in ***G***, if *g* is a *selective gram*, *g* is inserted into the index ***I*** with its occurrence information (Lines 9-10). We will explain the concept of the selective gram later. By using this concept, we can reduce the number of the grams to be indexed. Finally, IndexBuild returns the constructed indexes ***I_S_***, ***I_P_***, ***I_O_*** (Line 6).

#### Selecting indexing grams

In this section, we explain how we select the grams to be indexed. We use the gram selecting technique described in [12], where there are two goals for selecting grams: 1) maximizing the pruning power, and 2) minimizing the size of the index. To maximize the pruning power, the authors select infrequent grams that prune as many of the candidate strings as possible. To minimize the index size, they use the concept of the selective gram set. With this concept, they can further reduce the number of the grams to be indexed by removing redundant grams.

**Algorithm 1** IndexBuild

**Require:*D***; a set of triples

**Require:***α*, *β;* minimum and maximum size of a gram

**Ensure:** a set of indexes constructed {***I_S_***, ***I_P_***, ***I_O_***}

1: **for each** triple *t* in ***D***

2: {*S_S_*, *S_P_*, *S_O_*} *←* ExtractStrings(*t*);

3: ExtractAndInsertGrams(***I_S_***, *S_S_*, *α*, *β*);

4: ExtractAndInsertGrams(***I_P_***, *S_P_*, *α*, *β*);

5: ExtractAndInsertGrams(***I_O_***, *S_O_*, *α*, *β*);

6: **return** {***I_S_*, *I_P_*, *I_O_***};

**Function** ExtractAndInsertGrams

**Require:*I***; an index that extracted grams will be inserted

**Require:***S*; a string that grams will be extracted

**Require:***α*, *β*; minimum and maximum size of a gram

7: ***G*** ← FindAllGrams(*S*, *α*, *β*);

8: **for each** gram *g* in ***G***

9: **if***g* is a *selective gram***then**

10: insert *g* into the index ***I*** with its occurrence information;

To maximize the pruning power, [12] selects the grams which appear infrequently among all documents. The selectivity of the gram *g*, denoted as *Sel*(*g*), is defined as *C*(*g*)/*N*, where *N* is the total number of documents, and *C*(*g*) is the number of documents that contain the gram *g*. If the selectivity *Sel*(*g*) is low, the gram *g* appears infrequently. Thus, [12] can prune many candidates by using the gram *g* that has low selectivities. For example, for given grams *g*_1_ and *g*_2_, assume that the selectivities of these two grams are 0.1 and 0.9. They can prune 90% of documents using *g*_1_, while they can only prune 10% of documents using *g*_2_. To find the infrequent gram, for a given threshold *c*, they select grams where the selectivity of a selected gram is less than or equal to *c*, among all possible grams in all documents.

Even if only infrequent grams are selected, the number of grams remains large. Thus, [12] reduces the number of grams to be indexed further by removing the redundant grams. Assume that, for the string “GPR64_MOUSE”, the 3-gram “MOU” is an infrequent gram. Then, all the grams containing “MOU” are also infrequent grams. For example, “4_MOU”, “_MOU”, “MOUS”, and “MOUSE” are infrequent grams, because the selectivities of these grams are either less than or equal to *c*. Because these grams are redundant, it isn’t necessary to index all of them. Thus, they remove these redundant grams — the grams which contain another infrequent grams — in all infrequent grams. In this paper, we call the remaining grams selective grams.

### Index structure

In this section, we explain how to store and manage the selective grams. We use an inverted index for which the grams are the keys. Each key in the inverted index is associated with a posting list. The posting list contains occurrence information about the gram in the RDF database. Formally, for a given gram *g* and its occurrence information *p*, we insert a pair (*g*, *p*) into the index as the key/value. The occurrence information *p* is a set of (*tid*, *offs*), where *tid* is the ID of a triple containing the gram *g*, and *offs* is a set of offsets in the string where the gram *g* appears. Note that, g can appear many times in one string. The offsets in *offs* are used for the query processing as we will explain later. We also build a dictionary of all triples to convert a triple into the three-part string IDs. Using this dictionary, a triple ID in the posting list can be converted into a triple of string IDs.

In our framework, we construct three indexes for subject, predicate, and object parts separately, because the regular expressions for these three parts should be processed independently. That is, we construct three indexes, ***I_S_***, ***I_P_*** , and ***I_O_*** for each part of the RDF triples. Figure 2 shows an example of the inverted index that we construct. As the figure illustrates, each index of ***I_S_***, ***I_P_***, and ***I_O_*** has two data structures, a B^+^-tree and a posting list. The B^+^-tree stores the gram and the reference to the posting list as a key/value pair. Additionally, we also store the number of pages of the posting list in the value part. This information is used for the cost estimation of the query. The posting list contains the occurrence information. For example, the gram “MOUSE” appears in *t*_1_ and *t*_2_ at the sixth offset for both object part strings.

### Query processing

In this section, we explain the query processing algorithm. We first discuss the query processing without regular expressions for the background knowledge of the query processing in Section. Then, we explain how we process regular expression queries in Section.

#### Query processing without regular expressions

**Figure 3:**
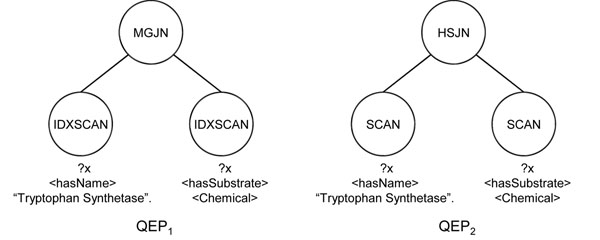
**An example of QEPs**. Query execution plans for the example query in Figure 1.

For a given SPARQL query *Q*, the query processor converts query *Q* into an optimal query execution plan (*QEP*), then executes the optimal QEP to get the results. The QEP is a rooted tree of operators. The leaf nodes of the QEP are scan operators associated with triples in the query *Q*. These scan operators find the triples in a database and return them to their parent nodes. The internal nodes of the QEP get the inputs from their child nodes, and they do the appropriate operations according to their operator types. *QEP*_1_ and *QEP*_2_ in Figure 3 are QEPs for the example query in Figure 1. The query in Figure 1 has two matching triples. The leaf operators of *QEP*_1_ and *QEP*_2_ are associated with these matching triples. These operators find the associated triples from the database, and return string IDs for the variable ?*x* to the parent operator MGJN (merge join) or HSJN (hash join). MGJN or HSJN operator gets the string IDs from child nodes, and returns the joining results of both inputs.

To find the optimal QEP, the query processor uses a query optimizer. The query optimizer finds the optimal QEP among all possible QEPs based on their costs computed by using a cost model. That means, the query optimizer enumerates all possible QEPs for a given query, and then selects the minimal cost QEP as the optimal QEP. For the query execution model, we use the GetNext model [30] which is very simple to use and has good scalability. Specifically, all operators in a QEP have GetNext() functions. For each call of the GetNext() function, the next results are returned one-by-one. To obtain all final results, the query executor calls the GetNext() function of the root operator of the optimal QEP until the function returns no more results. The GetNext() function calls are propagated to the descendants. That means the GetNext() function in the root operator calls the GetNext() functions of its child operators in order to get the results from them.

#### Query processing with regular expressions

To support regular expression queries, we develop a new operator, called *REGSCAN*, and adapt it to the query processing engine. For a triple pattern matching with a regular expression in a SPARQL query, the REGSCAN operator finds candidate triples which can be matched with that pattern in a database. In this section, we explain how this operator is created and implemented in a query execution.

### Plan generation

REGSCAN has a regular expression sub-plan to evaluate the regular expression. To generate this sub-plan, we adapt the technique in [12], and we summarize the technique in the next sub-plan generation section. We explain our technique using the example below. Here, the results for the query triple should contain substrings, in the object part, “GPR” or “CCD”, and following these substrings, the substring “MOUSE” must appear.

?*protein* <mnemonic> ?*m*. filter(?*m*,“(GPR|CCD).*MOUSE.*”)

**Figure 4:**
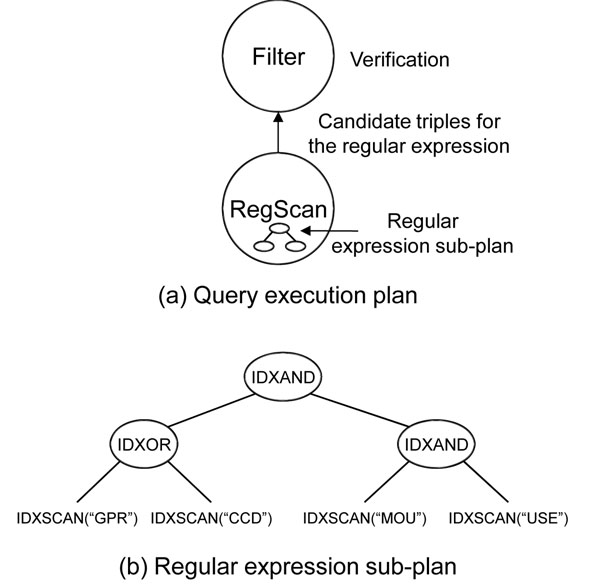
**An example of QEP with REGSCAN**. The QEP has two operators, FILTER and REGSCAN, in which REGSCAN contains regular-expression sub-plan

For the example above, we generate QEP with REGSCAN as in Figure 4(a). The QEP has two operators, REGSCAN and FILTER. The REGSCAN operator finds candidate triples for the matching triple pattern by using the containing-regular-expression sub-plan. Because the REGSCAN operator can find false positives, we must verify the results of the REGSCAN operator. The FILTER operator verifies the results from REGSCAN. We explain how the regular expression sub-plan in Figure 4(b) is constructed in the sub-plan generation part.

### Plan execution

The execution model of the regular expression sub-plan follows the GetNext model as well. Every sub-operator in the sub-plan has the GetNext() function, and it returns the results one-by-one for every GetNext() function call. The GetNext() function of the IDXSCAN for the gram *g* returns all triple IDs sequentially by scanning the posting list of *g*. IDXAND or IDXOR operators intersect or union the results from their children. The IDXAND operator uses offset information in the posting lists. When the offset of the triple from the left child is less than that of the right child and those triple IDs are the same, it returns the triple corresponding to this triple ID. When the IDXAND intersects two sets of the triple IDs, it uses this constraint.

The GetNext() function of REGSCAN operator calls the GetNext() function of the root operator of its sub-plan to get the candidate triple IDs and then converts each triple ID into three string IDs using the dictionary built in the index building algorithm. For example, REGSCAN operator in Figure 4(a) gets the triple ID by calling the GetNext() function of the root operator (IDXAND) of the regular expression sub-plan. Then, each triple ID is converted into the string IDs, and REGSCAN returns these string IDs to the FILTER operator to verify the result.

### Sub-plan generation

The technique in [12] has three steps for a given index ***I*** and a regular expression. These three steps are summarized as follows:

1. Normalize the input regular expression into the new regular expression that has only OR(*|*) or STAR(*).

2. Construct the parse tree using the normalized regular expression.

3. Convert the parse tree into the execution plan.

To adapt the technique in our framework, we first find the appropriate index among ***I_S_***, ***I_P_***, and ***I_O_*** by checking the position of regular expression appearing in the triple pattern. In the example query, the regular expression is bound with variable ?*m*, and ?*m* appears in the object part of the matching triple pattern. Therefore, we can select ***I_O_*** to evaluate the regular expression.

For the first step of the algorithm, the regular expression “(GPR *|* CCD). *MOUSE.*” in the query triple is converted into “(GPR *|* CCD)(a | b | c | …)*(MOUSE)(a | b | c | …)*”, after the normalization. Here, the periods(‘.’) are converted into the all characters connected with OR operators. In this step, for example, an aggregated regular expression, such as (a-z), is converted into (a | b | ··· | z), and a+ (more than one occurrence) is converted into aa*.

**Figure 5:**
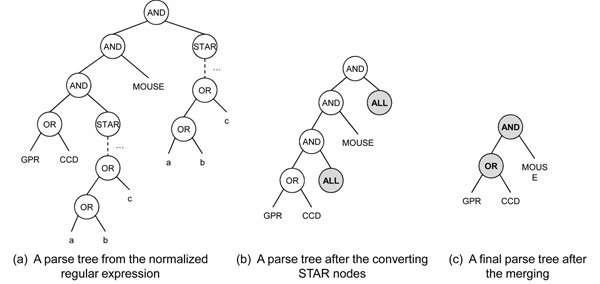
**An example of the parse tree building.** The parse tree is constructed from the normalized regular expression, STAR nodes converting, and ALL nodes merging.

In the second step of the algorithm, we can construct a parse tree using this normalized regular expression. The example of the parse tree for the normalized regular expression is represented in Figure 5(a). The leaf nodes in the parse tree contain grams that are separated by OR, AND, or STAR operators. Then, the sub-trees whose roots are the STAR nodes are converted into the ALL node. This is because STAR nodes can be represented as all combinations of their descendant nodes, and the number of possible grams for the STAR nodes is infinite. The shaded nodes in Figure 5(b) are the converted ones. After the conversion, the nodes that have the ALL nodes as their children are merged into AND or OR nodes with their children. If the node is an OR node and has an ALL node as its child, this node is merged into an ALL node. In this case, because the ALL node is generated again, the merging step is applied recursively, so that the final parse tree has only AND or OR nodes as its internal nodes.

**Figure 6:**
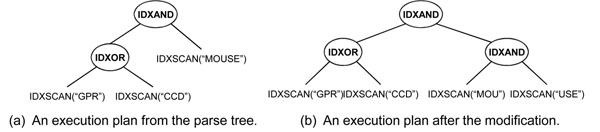
**An example of the regular expression sub-plan building.** An execution plan is built from the parse tree, and then modified according to the indexed grams.

In the third step, we build an execution plan using the parse tree constructed in the second step. In this step, we first convert the AND and OR nodes into IDXAND and IDXOR operators respectively, and leaf nodes are converted into IDXSCAN operators. An IDXSCAN operator for the gram *g* returns all IDs of the triples which contain *g*. An IDXAND or an IDXOR are logical AND or OR operators between the two sets of the triple IDs that come from its child operators. For example, Figure 6(a) represents the converted parse tree in Figure 5. The AND and OR nodes are converted into the IDXSCAN and IDXOR operators, and each leaf node *g* is converted into the IDXSCAN(*g*) operator.

Because all grams in the parse tree are not indexed, there may be no results for the IDXSCAN operators. Therefore, we need further modifications in the execution plan. For the gram *g*, there would be three cases: 1) *g* is indexed, 2) *g* is not indexed, but some substrings of *g* are indexed, and 3) *g* is not indexed, and its substrings are not indexed either. For the first case, we do nothing. In the second case, we replace the IDXSCAN(*g*) operators with the IDXAND operators of the IDXSCAN operators of *g*’s indexed substrings. In the third case, we replace the IDXSCAN node with the ALL node and apply the merging technique in the parse tree building step. For example, In Figure 6(b), because the gram “MOUSE” is not index, and its substrings “MOU” and “USE” are indexed, IDXSCAN(“MOUSE”) in Figure 6(a) is converted into the IDXAND between IDXSCAN(“MOU”) and IDXSCAN(“USE”).

### Cost model

In this section, we explain the cost model of the query processing. Our cost model is based on the cost of I/Os to access the index pages. The CPU cost for the verification of the candidate triples can also affect the execution time. However, we do not consider the CPU cost because it is negligible compared with the I/O cost. The cost for REGSCAN operator, *Cost*, is defined as follows:

*Cost* = *OnePageAccessCost* × (|*G*| × *Height*(***I***) + *∑_g∊G_ NumPages*(*g*)),

where *OnePageAccessCost* is a unit cost for accessing a page, *G* is a set of all grams in the execution plan, and *NumPages*(*g*) is the number of pages of the posting list for the gram *g*. To evaluate the execution plan, we have to access all posting lists and pages in the B^+^-tree.

This cost model can be used in the cost-based query optimizers of the RDF database. We exploit a cost-based bottom-up query optimizer to find the optimal query execution plan for the given SPARQL. Using the cost model, our algorithm can be adapted seamlessly.

## Results and discussion

### Setup

We implemented our framework using C++. The codes were compiled using the GNU gcc 4.3 compiler. All the experiments were conducted on a PC with an Intel Xeon Quad Core CPU and 8GB RAM running 64-bits Linux. We compared the performance of our algorithm with Sesame [22]. Our framework is denoted as RegScan. In our experiments, in order to avoid the problem of large database size as well as long index building time, we empirically select the values 2 and 4 for the parameters *α* and *β*, respectively. We use the threshold value 0.1, which is commonly used for finding infrequent grams for the parameter *c*. We used GeneOntolgy RDF dataset [17], a common real dataset which contains 869,770 triples for the experiments. For query processing, we used 8 queries which are presented in Appendix A. Before execution of each query, we flushed the OS caches using the /proc/sys/vm/drop_caches interface to minimize the caching effect.

### Results for the database building

Table 1 shows the database building time and the database size. In terms of the database building time, RegScan takes 4.57 times more time compared with Sesame, because RegScan has additional steps for the selective gram extraction. RegScan also needs 5.70 times more disk space compared with Sesame. This is because RegScan has to store additional indexes for scalable regular expression processing.

**Table 1:** **Database building time and database size** This table shows the database building time and the database size for RegScan and Seasame

	RegScan	Sesame
Time (sec.)	562.31	123.01
Size (MB)	532.69	93.48

### Results for the query processing

Table 2 shows the performance results for the GeneOntology dataset. According to this table, RegScan is the most effective among all algorithms. In terms of the query execution time, RegScan outperforms Sesame by up to 2143 times, and by 282 times on average.

**Table 2:** **Query execution time (sec.) for GeneOntology dataset** This table shows the execution time for 8 queries by using RegScan and Seasame

	RegScan	Sesame
Q1	0.01	11.91
Q2	0.04	12.53
Q3	0.01	21.43
Q4	0.02	12.50
Q5	0.04	3.94
Q6	0.02	3.77
Q7	0.02	3.60
Q8	0.11	6.41

This is because RegScan can avoid full-scanning of the database, and it only scans the posting lists which have very small sizes in comparison to the database size. We observe that RegScan performs more effectively, when the selectivity of the regular expression is low, since the regular expression with low selectivity accesses small sizes of posting lists for the grams. Since Sesame does not support scalable regular expression processing and is implemented using Java, it has much more overhead compared with our framework, and shows the worst performance results.

## Conclusions

In this paper, we have presented a novel framework which can be considered as the first solution for efficiently supporting regular expression processing of SPARQL queries over RDF databases. For building this framework, we first extract the grams from RDF data and efficiently build an inverted index structure based on the set of selective grams. Then, in the query processing, we find the indexed grams from regular expression patterns, and generate an efficient execution plan by using our proposed cost model for regular expression processing. To demonstrate the performance of our techniques, we have prototyped the proposed framework in C++ and then compared the efficiency and effectiveness of our systems with a popular RDF system (i.e., Sesame). The experimental results over large knowledge resources which are commonly used in bioinformatics, such as GeneOntolgy, have shown that our framework is an efficient and effective solution for processing regular expressions over RDF data and is useful for extracting information from bioinformatics knowledge databases.

## Authors' contributions

Jinsoo Lee took part in the implementation of the prototype system. He wrote the manuscript describing the methods and took part in conducting the experiments. Minh-Duc Pham wrote the remaining parts of the manuscript and conducted the experiments. Jihwan Lee implemented the prototype system with Jinsoo Lee. Wook-Shin Han developed the idea, supervised the work, and helped write the manuscripts. Hune Cho, Hwanjo Yu, and Jeong-Hoon Lee critically revised the manuscript. All authors read and approved the final manuscript.

## Competing interests

The authors declare that they have no competing interests.

## Appendix A: Query set for GeneOntology

**Q1.** SELECT * WHERE {?gp rdfs:label ?name FILTER regex (?name, ”spliceosomal”) .}

**Q2.** SELECT * WHERE {?gp rdfs:label ?name FILTER regex (?name, ”mRNA*|*tRNA”) .}

**Q3.** SELECT * WHERE {?gene ?pred ?isbn FILTER regex (?isbn, ”[0-9]19857[0-9]”) .}

**Q4.** SELECT * WHERE {?gp rdf:type ?type .?gp rdfs:label ?name FILTER regex (?name, ”BP.*mouse”) .}

**Q5.** SELECT * WHERE {?gp rdf:type ?type FILTER regex (?gp, ”0005[0-9][0-9][0-9]”) .?gp rdfs:label ?name .?gp rdfs:comment ?comment .}

**Q6.** SELECT * WHERE {?gp rdf:type ?type FILTER regex (?gp, ”0005[0-9][0-9][0-9]”) .?gp rdfs:label ?name FILTER regex (?name, ”activity”) .?gp rdfs:comment ?comment .}

**Q7.** SELECT * WHERE {?gp rdf:type ?type FILTER regex (?gp, ”0005[0-9][0-9][0-9]”) .?gp rdfs:label ?name FILTER regex (?name, ”activity”) .?gp rdfs:comment ?comment FILTER regex (?comment, ”molecular”).}

**Q8.** SELECT * WHERE {?gp1 go:consider ?gp2 . ?gp1 ?p1 ?ns1 FILTER regex (?ns1, ”cellular”) .?gp2 ?p2 ?ns2 FILTER regex (?ns2, ”molecular”) .}
